# Supplementary material for: Telemedicine management of type 2 diabetes mellitus in obese and overweight young and middle-aged patients during COVID-19 outbreak: A single-center, prospective, randomized control study
Source: PLoS One. 2022 Sep 29;17(9):e0275251. doi: 10.1371/journal.pone.0275251 (PMC9522303; doi:10.1371/journal.pone.0275251)
Supplement: S1 Protocol — (PDF) [file pone.0275251.s002.pdf]

ClinicalTrials.gov PRS
Protocol Registration and Results System

ClinicalTrials.gov PRS DRAFT Receipt (Working Version)
Last Update: 04/28/2022 19:21

ClinicalTrials.gov ID: NCT04723550

Study Identification

Unique Protocol ID: NO.2020QN80

Brief Title: COVID-19 Lockdown Related Telemedicine for Type 2 Diabetes

Official Title: Impact of Telemedicine on Young and Middle-aged Obese Patients With Type 2 Diabetes Mellitus During COVID-19 Pandemic

Secondary IDs:

Study Status

Record Verification: January 2021

Overall Status: Completed

Study Start: January 5, 2021 [Actual]

Primary Completion: December 5, 2021 [Actual]

Study Completion: February 5, 2022 [Actual]

Sponsor/Collaborators

Sponsor: Wenwen Yin

Responsible Party: Sponsor-Investigator
Investigator: Wenwen Yin [wyin]
Official Title: M.D.
Affiliation: Xuzhou No.1 Peoples Hospital

Collaborators:

Oversight

U.S. FDA-regulated Drug: No

U.S. FDA-regulated Device: No

U.S. FDA IND/IDE: No

Human Subjects Review: Board Status: Approved
Approval Number: xyy11[2020]40
Board Name: Medical Ethics Committee of Xuzhou No.1 Peoples Hospital
Board Affiliation: Xuzhou No.1 Peoples Hospital
Phone: +86(0516)68167579
Email: fengfangxiaoyu@163.com
Address:

269 Daxue Road, Tongshan District,Xuzhou,China.

Data Monitoring: No

FDA Regulated Intervention: No

Study Description

Brief Summary: At present, in order to cope with the global pandemic of the COVID-19 virus, governments have introduced corresponding measures, COVID-19 lockdown is one of the most important measures. However, lockdown makes the management of chronic diseases (such as type 2 diabetes) more difficult, and telemedicine may be one of the solutions. We hope to explore the effect of telemedicine on blood glucose control and other prognostic indicators of young and middle-aged obese patients with type 2 diabetes who will experience isolation control.

Detailed Description: We recruit patients with type 2 diabetes who need to be isolated due to the COVID-19 epidemic, Our study will include young and middle-aged obese patients. The lockdown period is 21 days. The patients will be randomly divided into two groups with a total follow-up time of 6 months. One group is the telemedicine intervention group, and the other group is the routine follow-up control group. The intervention group used the hospital telemedicine management system to upload blood glucose values (fasting and 2h after three meals), food intake of three meals, and exercise volume (Data collection frequency: first three months, 4 times/week; 4-6 Month, 2 times/week).Doctors will collect data from hospital telemedicine management system to guide patients on diets, exercise, and medication adjustments. The control group will be followed up by telephone/outpatient clinic every 1 week. (only telephone follow-up will be conducted during the lockdown period) Then doctors will collect their blood glucose values.(fasting and 2h after three meals) Based on the data collected, The doctors will provide lifestyle guidance to the patients on the telephone or face to face. The clinical data of the two groups of patients will be collected at baseline, 22 days, 3 months, and 6 months respectively. (HbA1c, fasting blood glucose(FBG), blood glucose 2 hours after breakfast, blood pressure, Body Mass Index(BMI), waist-to-hip ratio, total cholesterol(TC), triglyceride(TG), high-density lipoprotein cholesterol(HDL-C), low-density lipoprotein cholesterol(LDL-C), Blood Urea Nitrogen(BUN), serum creatinine(Scr), e-GFR, Self-rating Depression Scale, frequency of hypoglycemia#and Cost effectiveness) The clinical data will be statistically analyzed.

Conditions

Conditions: Type 2 Diabetes Mellitus

Keywords: Type 2 Diabetes Mellitus
COVID-19 lockdown
Telemedicine
Obesity
Young and middle-aged

Study Design

Study Type: Interventional

Primary Purpose: Health Services Research

Study Phase: N/A

Interventional Study Model: Parallel Assignment

Number of Arms:	2
Masking:	None (Open Label)
Allocation:	Randomized
Enrollment:	99 [Actual]

Arms and Interventions

Arms	Assigned Interventions
Experimental: Telemedicine<br>Diabetes education and support by telemedicine	Device: Hospital telemedicine management system<br>Patients upload data of blood glucose, diet and exercise. Then doctors guide patients' diet, exercise and medication adjustment through the telemedicine system.
Active Comparator: Usual care<br>Diabetes education and support in person	Usual care<br>Outpatient/telephone follow-up#continued care, as usual, from their primary care provider through out duration of action 6 months intervention period

Outcome Measures

Primary Outcome Measure:

1. Glucose control (HbA1c levels)
   Change in HbA1c among control and telemedicine groups from baseline to 6 months

   [Time Frame: Baseline, 22days,3 months and 6 months]

Secondary Outcome Measure:

2. Change in FBG
   Change in FBG among control and telemedicine groups from baseline to 6 months

   [Time Frame: Baseline, 22days,3 months and 6 months]
3. Change in Blood glucose 2 hours after breakfast
   Change in Blood glucose 2 hours after breakfast among control and telemedicine groups from baseline to 6 months

   [Time Frame: Baseline, 22days,3 months and 6 months]
4. Change in Blood pressure
   Change in Blood pressure among control and telemedicine groups from baseline to 6 months

   [Time Frame: Baseline, 22days,3 months and 6 months]
5. Body mass BMI changes
   Comparison of BMI changes among control and telemedicine groups from baseline to 6 months

   [Time Frame: Baseline, 22days,3 months and 6 months]
6. Change in waist-to-hip ratio
   Comparison of waist-to-hip ratio changes among control and telemedicine groups from baseline to 6 months

   [Time Frame: 6 months]
7. Change in biological parameter: TC
   Variation between baseline to 6 months of Biological parameter among control and telemedicine groups: TC

   [Time Frame: Baseline, 22days,3 months and 6 months]
8. Change in biological parameter: TG
   Variation between baseline to 6 months of Biological parameter among control and telemedicine groups: TG

[Time Frame: Baseline, 22days,3 months and 6 months]

9. Change in biological parameter: HDL-C
   Variation between baseline to 6 months of Biological parameter among control and telemedicine groups: HDL-C

   [Time Frame: Baseline, 22days,3 months and 6 months]

10. Change in biological parameter: BUN
    Variation between baseline to 6 months of Biological parameter among control and telemedicine groups: BUN

    [Time Frame: Baseline, 22days,3 months and 6 months]

11. Change in biological parameter: Scr
    Variation between baseline to 6 months of Biological parameter among control and telemedicine groups: Scr

    [Time Frame: Baseline, 22days,3 months and 6 months]

12. Change in biological parameter: e-GFR
    Variation between baseline to 6 months of Biological parameter among control and telemedicine groups: e-GFR

    [Time Frame: Baseline, 22days,3 months and 6 months]

Other Pre-specified Outcome Measures:

13. Change in scores measured by Self-rating Depression Scale
    Self-rating Depression Scale includes 20 items in four dimensions of psychological disorders, namely, psychotic emotional symptoms, somatic disorders, psychomotor disorders, and depression. The maximum value of SDS is 50 points. A lower total score means a better situation in terms of depression and vice versa
    [Time Frame: Baseline, 22days,3 months and 6 months]

14. Number of hypoglycemia events
    Hypoglycemia events for telemedicine group versus control group
    [Time Frame: 6 months]

Eligibility

Minimum Age: 18 Years

Maximum Age: 55 Years

Sex: All

Gender Based:

Accepts Healthy Volunteers: No

Criteria: Inclusion Criteria:

- Physician diagnosis of Type 2 diabetes for more than 6 months
- 7.0%#HbA1c#10.0%
- Quarantine for 21 days due to COVID-19 outbreak related reasons
- age: 18 ~ 55 yrs
- BMI≥24
- Be able use smart phones and the Internet

Exclusion Criteria:

- Insulin pump users
- For female subjects: pregnancy or lactation, or subject may become pregnant during the study

- Patient who underwent obesity surgery to the exclusion of a gastric band, loosened or removed for more than a year
- Patients diagnosed with COVID-19 infection
- Have severe complications (chronic heart disease, cerebrovascular disease, diagnosed HIV/AIDS, cancer, emphysema, chronic liver or kidney disease) that would affect the subjects' ability to follow the tailored advice

Contacts/Locations

Central Contact Person: Wenwen Yin, MD.
Telephone: +86 18112008016
Email: wenwen261621@163.com

Central Contact Backup: Ning Ding, MD.
Telephone: +86 17712987026
Email: 961897477@qq.com

Study Officials: NOTE : Study Official is required by the WHO and ICMJE.

Locations: **China, Jiangsu**
Department of Endocrinology, Xuzhou NO.1 Peoples Hospital
Xuzhou, Jiangsu, China, 221000
Contact: Wenwen Yin +86 18112008016 wenwen261621@163.com

IPDSharing

Plan to Share IPD:

References

Citations:

Links:

Available IPD/Information:
