# Supplementary material for: Telemedicine management of type 2 diabetes mellitus in obese and overweight young and middle-aged patients during COVID-19 outbreak: A single-center, prospective, randomized control study
Source: PLoS One. 2022 Sep 29;17(9):e0275251. doi: 10.1371/journal.pone.0275251 (PMC9522303; doi:10.1371/journal.pone.0275251)
Supplement: Attachment — Submitted filename: Response to Reviewers.docx [file pone.0275251.s003.docx]

**Responds to the reviewer’s comments:**

**Review1#**

- **Response to comment:** Line 81-82: “to help the patients to achieve blood glucose control”. I understand that the paper mainly focusses on glycaemic control, however this sentence is reductive, since, in diabetic patients, the real benefit is provided by the multifactorial control rather than one (doi: 10.1186/s12933-021-01343-1).

**Response:** First of all, thank you very much for reviewing our article and giving a fair review of the comments. Your suggestion is correct and reasonable. As you said, "In diabetic patients, the real benefit is provided by the multifactorial control!" Using telemedicine, we hope to manage diabetes mellitus as comprehensively as possible, in the context of COVID-19 quarantine measures. The measures in our study focused on the popularization of diabetes-related knowledge, diet and exercise guidance, and adjustment of blood glucose monitoring and glucose-lowering regimens. The outcome was satisfying. So, as you said, the statement in the original text is glaringly partial. Thanks for your reminder, we have deleted (Original page 3 Line81-82)"and help patients achieve blood glucose control", and added relevant contents in the Discussion (Page22 Line 385- 390)"In recent years, the role of multifaceted management in patients with T2DM has been repeatedly emphasized [37]. The telemedicine measures provided by this study focused more on the popularization of diabetes mellitus-related knowledge, diet/exercise guidance, blood glucose monitoring, and adjustment of glucose-lowering regimen. We believe that patients should receive more comprehensive multifactorial control if conditions permit. Further research is warranted in this regard. "

- **Response to comment:** Moreover, telemedicine as a screening tool has proven its efficacy both during covid pandemic and before, not representing a novel option (doi: 10.1155/2020/9036847; doi: 10.1002/dmrr.3113).

**Response:** As you said, "telemedicine as a screening tool has proven its efficacy both during the Covid-19 pandemic and before" We also mentioned in the original text (Original page 18 Line303-304)"At present, many studies on telemedicine management for diabetes have reported favorable outcomes," (Original page 5 Line79-81)"Arabi et al.'s study showed that telemedicine had positive significance for blood glucose control during COVID-19 pandemic isolation," and we found that (Original page 5 Line81-85)"It is noteworthy that the sudden lifestyle changes, in terms of a cessation of outdoor movement and social interaction, that occurred during the COVID-19 lockdown, had a great impact on the young and middle-aged population, which may have led to physical and mental diseases, such as obesity, depression, and anxiety [11, 12]." So our research focuses on this group of people. Of course your advice is so critical, we have deleted (Original page 11)" but the relevant research during the COVID-19 pandemic is relatively rare, and ", and put the references you provided in the introduction.

- **Response to comment:** As this is a clinical trial, missing patients should be less than 5%. In this specific case 120 patients were enrolled and only 99 finished the period of follow up (missing 17.5%). The problem of missing data is of particular importance due to it introducing bias and leading to a loss of power, inefficiencies, and false positive findings (Type I Error). It MUST be reported in the limit of the study. Moreover, it should be reported in the appropriate section what happened to these patients (retrieved consent, etc…).

**Response:** Thanks for the reminder, we have added the issue you mentioned in the Discussion (Page22 Line395-398)"Third, the authors must acknowledge the shortcomings of missing data in this study. In this specific case, 120 patients were enrolled and only 99 finished the period of follow-up (missing 17.5%). Due to missing data, the findings of this study should be considered with caution. "

- **Response to comment:** Moreover, it should be reported in the appropriate section what happened to these patients (retrieved consent, etc…).

**Response:** (Page10 Line186-189) "Of the 201 potentially eligible participants, 120 participants expressed interest and were screened for suitability for inclusion – of these, six in the intervention group and 11 in the control group voluntarily withdrew from the study, and two in each group were lost to follow-up. "

- **Response to comment:** Discussion: it has been pointed out that depression may affect diabetes. Another important element is the environment. In fact, family dietary habit, patients dietary habit (the patients where isolated at home for 21 days, but most of them may be consumers of junk food during daytime due to work) etc… should be also taken into consideration.

**Response:** As you said, the environment also has a critical role in diabetes. At the end of the isolation period, many outpatient visits complained of low activity, excessive television viewing, and junk food intake when isolated at home. This has been noted in our Discussion also (Original page 20 Line343-349)"Both groups were able to receive diet and exercise instructions; however, the medical team including doctors, nurses, and nutritionists, could more frequently monitor the diet of patients in the intervention group through telemedicine, allowing for more precise guidance. Strategies such as frequent weighing, recording energy intake, and tracking physical activity are currently used to improve compliance, but the above strategies are insufficient to produce significant weight loss without feedback[29]." In this context, the present findings deserve special attention. Based on your suggestion, we add the following to the Discussion (Page20 Line354-359)"In addition, the fast food consumed by young and middle-aged people during their daily work is often calorie-rich junk food. During the 21-day isolation and under expert dietary guidance, the intervention group participants were able to improve their dietary structure, which may also be one of the reasons for the efficient glycemic and weight control in these patients. "

**Reviewer2#**

- **Response to comment:** It is critical to calculate the sample size when you design the study, especially for the confirmatory analysis of the effects. This is a major flaw of the study.

**Response:** Thank you very much for your reminder, your advice is of critical importance. At the experimental design stage, we performed sample size calculations. We've added this part to Methods.

Pre-experimental results:

In the pilot experiment, HbA1C was used as the outcome indicator. The intervention group was the telemedicine group, and the control group was the outpatient follow-up group, with 10 people in each group. The HbA1C reduction rate was 2.41% (standard deviation 1.38%) in the outpatient group and 1.67% (SD 1.15%) in the telemedicine group after 6 months of follow-up respectively. Set the bilateral α = 0.05, the power of the test is 80%, and the sample size of the control group: experimental group = 1: 1. Using PASS 15.0 (NCSS LLC) software, the sample size of the intervention group and the control group was 48.

Figure 1 Sample size calculation results

If 20% of the cases of loss of follow-up and refusal of follow-up are considered, a sample size of 60 patients in each group is required. The total sample size was 120 cases.

Figure 2 Sample size considering 20% loss of follow-up

We have added this part in Mehtods：

(Page8 Line160-168)

**Sample size**

A previous observational study including 10 patients described a 2.41% (standard deviation, SD = 1.38%) reduction in the HbA1c value in the outpatient group and 1.67% (SD = 1.15%) in the telemedicine group after 6 months of follow-up. Based on these results, using a bilateral α = 0.05 to achieve 80% test power and an equal ratio of patient allocation to the control and experimental groups (1:1), we computed a target sample size of 48 subjects in each group using PASS 15.0 (NCSS LLC). Further, accounting for a 20% loss of sample to follow-up or refusal to follow-up, a sample size of 60 patients in each group was decided.

- **Response to comment:** The statistical analysis is under development. For the repeated measures, better to use longitudinal models.
- **Response to comment:** P values need to be adjusted for multiple tests. How many tests will lose significance after adjustment? The conclusions should be re-evaluated after correct statistics are performed.

**Response:** Your advice is highly important, thank you very much for your guidance! We have re-statistics the experimental data to obtain more convincing results.

(Page9Line179-183) "The primary outcome, HbA1c, was evaluated at the four aforementioned time points using two-way repeated-measures ANOVA. The group effect, time effect, and interaction of group and time (group*time) were compared between groups and within groups. Bonferroni correction was adopted for post-hoc comparison. A p-value of <0.05 was considered statistically significant for all analyses. "

(Page11Line225-240)

Trends in the HbA1c values during the follow-up (Table 3)

The two-way repeated-measures ANOVA found that the main effect of the group was not significant (F = 2.558, p = 0.113, partial η2 = 0.026), whereas, there was a significant main effect of time (F = 86.089, p < 0.001, partial η2 = 0.470) and the interaction between group and time (F = 3.498, p = 0.019, partial η2 = 0.035). The separate group effect was not significant at baseline (F = 0.106, p = 0.746, partial η2 = 0.001) and at the end of the isolation period (F = 0.361, p = 0.55, partial η2 = 0.004. However, at the end of 3 months, the simple effect of the group was significant (F = 5.765, p = 0.018, partial η2 = 0.056), which again became non-significant at the end of 6 months (F = 3.659, p = 0.059, partial η2 = 0.036). The simple effect of time was significant both in the intervention group (F = 59.184, p < 0.001, partial η2 = 0.651) and the control group (F = 29.001, p < 0.001, partial η2 = 0.478). Post-hoc comparisons showed that in the intervention group, HbA1c was lower at 3 and 6 months than at baseline and at end of isolation (p < 0.05), whereas in the control group, the HbA1c value at the end of 6 months was lowest among all four values for this group (p < 0.05).

Table 3. A comparison of the primary outcome, HbA1c, throughout the 6-month follow-up

|  | n | Baseline | 21 days | 3 months | 6 months | *F* | *P* | Partial *η*^2^ |
| --- | --- | --- | --- | --- | --- | --- | --- | --- |
| Intervention | 52 | 8.56 ± 0.88 | 8.57 ± 1.43 | 6.60 ± 1.31^ab^ | 6.14 ± 1.05^ab^ |  |  |  |
| Control | 47 | 8.50 ± 0.80 | 8.41 ± 1.21 | 7.34 ± 1.71^ab^ | 6.66 ± 1.63^abc^ |  |  |  |
| Group main effect |  |  |  |  |  | 2.558 | 0.113 | 0.026 |
| Time main effect |  |  |  |  |  | 86.089 | <0.001 | 0.470 |
| Group*Time interaction effect |  |  |  |  |  | 3.498 | 0.019 | 0.035 |

Compared with baseline, ap < 0.05; compared with 21 days, b p < 0.05; compared with the end of 3 months, c p < 0.05.

- **Response to comment:** T-test was mentioned but was not used.
- **Response to comment:** Figure 2 It is not clear whether the bars are means or medians. How about the error bars? Better be consistent with Table 2. If you decide to use median, then present medians in the table and in the figure. Do not mix usage of parametric and nonparametric methods.

**Response:** Thank you for your reminder. We have made corrections to avoid misunderstanding. (Page9Line171-173) "Normally distributed variables are expressed as means and SD, and non-normally distributed variables are expressed as the median and interquartile range (IQR)." At the same time, we have modified Figure 1 and 2 to make the data presentation stay in line with Table.

- **Response to comment:** Is should be Wilcoxon signed…

**Response:** Thank you very much for your guidance, we have corrected the mistake.

- **Response to comment:** Minor concerns
- **Response to comment:** Table 1 & 2 add p values for comparisons

**Response:** Thanks for the reminder, we have added the P value of intergroup comparison to the table.

- **Response to comment:** It is better to show reduction from baseline in table 2.

**Response:** Thank you for the reminder! We have added the "Reduction" to the table.

- **Response to comment:** Page 7. Add space between medians and brackets, and space after coma.

**Response:** Thank you very much for your nuanced advice, we have made changes!

- **Response to comment:** Figure 1 how are the participants stratified? “Analyzed date” is not correct.

**Response:** We are very sorry, this is a clerical error, we have corrected it and this study did not stratify the patients. In addition, you pointed out that “Analyzed date” is not correct, we have corrected “Analyzed date” to “Available for analysis”

- **Response to comment:** The writing needs to be polished by a native speaker.

**Response:** Thank you for your comments. We have asked professionals to polish the full text. We put the “**CERTIFICATE OF ENGLISH EDITING**” at the end of this article.

**Review3#**

- **Response to comment:** In the text, less has been reported about the cellular mechanisms of viral entrance, replication and pathogenesis, and more less data about the clinical outcomes of COVID-19 in patients at higher risk as reported for patients with hypertension and type 2 diabetes mellitus. In this setting, I would remember:
- **Response to comment:** -in the pathogenesis of SARS/COV2 infection, the role played by serin proteasis expression (TMPRSS2) in humans’ cells as main cause of the entrance and replication of SARS/COV2 (miR-98 Regulates TMPRSS2 Expression in Human Endothelial Cells: Key Implications for COVID-19. Biomedicines. 2020 Oct 30;8(11):462. doi: 10.3390/biomedicines8110462), and parallel the different cellular expression of ACE2 in humans, and its negative effects on clinical outcomes in humans (Cardiovasc Diabetol. 2021 May 7;20(1):99. doi: 10.1186/s12933-021-01286-7). Please describe this point and refer to the suggested reference.

**Response:** Thank you very much for your detailed comments and suggestions. We apologize for not explaining our research clearly. Our study population was recruited from all T2DM patients who had been admitted to our center before. as mentioned in the Methods (Original page 6 Line101-103)" For patients with T2DM who were seen in our outpatient endocrine clinic before, we conducted a telephone follow-up interview. These patients were candidates forthis randomized controlled trial." China's current epidemic prevention policy for the COVID-19 is still "dynamic zero-Covid policy". If there's a positive patient somewhere, the government will take control and lock-down measures in a large area, which also provides conditions for our study. Strict control measures have enabled the epidemic to be quickly brought under control, and the incidence is extremely low in the population. In our study, multiple throat swabs were collected over time as required by the epidemic prevention policy. However, at the end of the study, no COVID-19 positive cases were found in all the study cases. Of course, the purpose of this study was to explore the application of telemedicine to manage obese/overweight young and middle-aged T2DM patients during the epidemic. Your suggestion has attracted enough attention from us! We suspect that even in China, the outcome of COVID-19 should be herd immunity. The mechanism underlying the association between COVID-19 and hypertension/diabetes will be further studied at that time. To avoid misunderstanding, we add the following statement to Results (Page10 Line190-192)" According to COVID-19 diagnostic criteria, no confirmed cases of COVID-19 were found in this study [15, 16]." Following your suggestions, we briefly described the relevant molecular mechanisms of COVID-19 infection in T2DM patients in the Discussion, which will be listed below.

- **Response to comment:** -please introduce the population at higher risk of worse prognosis as the hypertensive patients (Could anti-hypertensive drug therapy affect the clinical prognosis of hypertensive patients with COVID-19 infection? Data from centers of southern Italy. J Am Heart Assoc. 2020 Jul 7:e016948. doi: 10.1161/JAHA.120.016948), and the diabetics (Outcomes in Patients With Hyperglycemia Affected by COVID-19: Can We Do More on Glycemic Control? Diabetes Care. 2020 Jul;43(7):1408-1415. doi: 10.2337/dc20-0723; Impact of diabetes mellitus on clinical outcomes in patients affected by Covid-19. Cardiovasc Diabetol. 2020 Jun 11;19(1):76. doi: 10.1186/s12933-020-01047-y). Indeed, these patients are those at higher risk for COVID19, ICU admission and deaths. Please explain this point in the text, and the association between these cohorts of patients and the worse prognosis. What is your opinion? Please discuss it.

**Response:** We really appreciate your suggestions, but this study did not focus on the observation of hypertension, so we did not discuss the relationship between hypertension and COVID-19 infection. We added in the Introduction (Page4 Line69-70) "In addition, diabetic patients are more susceptible to contracting the COVID-19 infection and have a worse prognosis [5-7]." In the original Discussion, we have already mentioned (Original page 17 Line280-282) "Diabetic patients are more susceptible to COVID-19 and reportedly have a rapid disease progression, high severity, and high fatality rate after infection [17]." Based on your suggestion, we have added the following to the Discussion:

(Page17 Line282-294)

"It is believed that the upregulation of the angiotensin-converting enzyme (ACE)-2 receptor expression in cardiomyocytes of patients with diabetes mellitus, along with nonenzymatic glycation, may increase the susceptibility to COVID-19 infection in diabetic patients by promoting SARS-CoV2 cell entry [18]. Furthermore, a diabetic patient’s immune dysfunction, proinflammatory cytokine environment, hypoglycemic state, and coagulopathy contribute to a poor prognosis and complications of COVID-19 by increasing the risk of mechanical ventilation, shock, and multiple organ failure and death [19-23]. Sardu showed that early glycemic control reduces adverse outcomes in hospitalized hyperglycemic COVID-19 patients with or without a previous diagnosis of diabetes [23]. Therefore, ensuring competent diabetic management and taking scientific and effective protective measures during the pandemic is crucial to improving immunity and reducing the risk of infection in diabetic patients."

METHODS:

- **Response to comment:** -How did you diagnose T2DM?

**Response:** We have added relevant references to Methods. We employed WHO (1999) Diagnostic criteria to diagnose diabetes. [1]

- **Response to comment:** what were the cut off Hb1Ac values for inclusion/exclusion study criteria?

**Response:** We have indicated in the inclusion criteria of Participants and Recruitment in the Methods of the original article: (Page6 Line105) "7.0%＜HbA1c＜10.0%."

- **Response to comment:** -report the full data about the industry implied/used for laboratory analysis.

**Response:** Thank you very much for your reminder, we have uploaded the relevant data to the ClinicalTrail database.

- **Response to comment:** Report in detail study population, inclusion vs. exclusion criteria.

**Response:** Please allow me to explain to you that we have elaborated this section in the original Methods-- Study participants and recruitment (Page6 Line99). Thank you for your attention!

- **Response to comment:**-How did you diagnose and monitor study endpoints? Please discuss it, including all techniques and methods for measuring the study outcomes.

**Response:** Thank you very much for your question and we would like to explain it to you again. We followed up with the enrolled patients for six months and collected clinical data. At the end of this study, no COVID-19-positive patients were found. This study does not involve death, cure, recurrence, and other outcomes. Of course, in the design of the trial, we also mentioned withdrawal from the study if the patient developed serious complications and was unable to complete the trial. This did not happen. All the indicators involved in this test can be obtained by a laboratory tests, such as HbA1C, BP, TG, TC, etc. The measurement method of the Self-rated Depression Scale（SDS） has been described in the original Method. RT-PCR nucleic acid detection is an important method for diagnosing SARS-CoV and MERS-CoV, with high specificity and sensitivity, [2,3] and it is also the laboratory detection method for COVID-19 in our unit.

- **Response to comment:** You wrote that “Data is presented as the median (Quartile 1-Quartile 3) or the number of participants (%)”. It does not look. It looks as the data is presented as mean ± standard deviations and/or as median values. Please correct and discuss it.

**Response:** Thank you very much for your reminder. To avoid misunderstanding, we have made modifications, including the description of Statistical analysis in Methods, as well as Table and Figure, to make all data representations consistent.

- **Response to comment:** Please include all the measures units of study variables. The same in tables.

**Response:** Thanks for your suggestion, we have made changes.

- **Response to comment:** The study cohorts are represented by a low number of subjects. In my opinion the statistics could be under powered, and this limiting the study results. How did you calculate the sample size? Please address the question.

**Response:** At the experimental design stage, we performed sample size calculations. I'm sorry for missing this issue before. Thanks to your reminder, we've added a description of the sample size calculation to Methods (Page8 Line160-168).

- **Response to comment:** Tables and figures are of poor quality. Improve it.

**Response:** Thanks for the tip, we've modified the Table and Figure and added data for easy reading.

- **Response to comment:** The legend of figure 2 is not clear. What does it mean “a P﹤0.05 ,b P<0.01; between–group comparisons, * P<0.05, ** P<0.01”. I do not understand. Could it be: * p<0.05 at baseline; ** p<0.05 at follow-up end? Please respond and report the right correction.

**Response:** We are very sorry that our previous expression was biased and caused misunderstanding, and we have revised this part of the expression. (Page12 Line247-249) “a” indicates p < 0.05 versus baseline; “b” indicates p < 0.01 versus baseline; “*” indicates p < 0.05 versus control group; “**” indicates p < 0.05 versus the control group.

- **Response to comment:** Please add the column with p values by comparing cohorts of study.

**Response:** Thanks for the suggestion, we have added the P value in Table 2.

- **Response to comment:** Add the full medical therapy as anti-SARS-CoV-2, and anti-diabetic medications. Could it affect clinical study outcomes? Please discuss it.

**Response:** Thank you for your question. As mentioned above, we did not find COVID-19-positive patients in our study and therefore did not use anti-SARS-CoV-2 drugs. We guide diabetes mellitus patients to medication according to "Standards of Medical Care in Diabetes".[4] During the study, 10 subjects in the control group and 6 subjects in the intervention group required adjustment of drug dosage for titration of glycemic control; And as we described in the original Discussion (Original page 19 Line323-327) " Also, the intervention group had significantly fewer hypoglycemic events compared with the control group (n = 8 vs. n = 13). This difference may be attributed to the fact that the medical team was able to timely identify the risk of hypoglycemia in the intervention group through telemedicine and take appropriate measures. "

- **Response to comment:** how many patients were under insulin therapy in the cohorts during hospitalizations?

**Response:** At the beginning of the study, the rate of insulin use in the intervention group was 17.3% (9 cases) and 17.02% (8 cases) in the control group. At the end of 6 months, insulin use was 7.7% (4 patients) in the intervention group and 10.6% (5 patients) in the control group. Ultimately, there were no confirmed COVID-19 positive patients in the study population.

- **Response to comment:** what was the rate of anti-diabetic medications discontinuation during hospitalization? What was the mean glycemia during hospital discharge?

**Response:** Thank you for your question. As mentioned earlier, we studied patients who were isolated at home due to the government's epidemic prevention policy. The lock-down period was 21 days and the total follow-up period of the study was 6 months. At the end of 6 months, the rate of anti-diabetic medications discontinuation in the intervention and control groups was 11.5 vs 2.1, respectively. The glucose values of the two groups at the end of 6 months are shown in Table 2.

- **Response to comment:** what were the patients under anti-IL6 (tolicizumab) therapy? Indeed, also in treated patients there could be a loss of effect caused by the negative factors as hyperglycemia (Negative impact of hyperglycaemia on tocilizumab therapy in Covid-19 patients. Diabetes and Metabolism 2020; doi: 10.1016/j.diabet.2020.05.005). please discuss this point and the suggested reference.

**Response:** Thank you for your question. There are no confirmed COVID-19 patients in our study, so anti-IL6 therapy is not available.

- **Response to comment:** Indeed, the hyperglycemia is a recognized and reported factor of worse prognosis in the patients with COVID-19 (Hyperglycaemia on admission to hospital and COVID-19. Diabetologia. 2020 Jul 6:1-2. doi: 10.1007/s00125-020-05216-2). Please discuss and clarify this point in the text.

**Response:** Thanks for the reminder, we've added relevant content to the discussion based on your suggestion. The reference you provided is very important. We have quoted it. Thank you again!

- **Response to comment:** Finally, there are not data about the vaccinated patients under hyperglycemia and the COVID-19. Notably, again the hyperglycemia could reduce the efficacy of vaccination (The CAVEAT study. Diabetes Obes Metab. 2022 Jan;24(1):160-165. doi: 10.1111/dom.14547; Nat Commun. 2022 Apr 28;13(1):2318. doi: 10.1038/s41467-022-30068-2). Please discuss it.

**Response:** Thank you for your advice! At the beginning of the study, the vaccination rate was 73% in the control group and 76% in the intervention group. Because our study did not involve confirmed COVID-19 patients, we did not include this indicator in this study.

- **Response to comment:** DISCUSSION:
- **Response to comment:** It is too long, and not well focused on main study outcomes. Please rewrite it.

**Response:** The authors are grateful to you for the valuable comments and suggestions! In the Discussion of this paper, we discussed the experimental results in three parts, namely: 1. Telemedicine contributes to blood glucose management in young and middle-aged type 2 diabetes mellitus patients with obesity/overweight during COVID-19; 2. Telemedicine is helpful for weight control of young and middle-aged type 2 diabetes patients with type 2 diabetes who are obesity/overweight during the COVID-19 epidemic; 3. During the COVID-19 pandemic, telemedicine appears beneficial in reducing symptoms of depression in young and middle-aged type 2 diabetes patients who are obese/overweight. The research priorities were carefully elaborated by the three parts above. Your suggestion is so important to us, and provides a more in-depth understanding of the correlation between T2DM and COVID-19. We have added your advice to the Discussion. Our future work should further examine this issue.

**References**

1.	Alberti KG, Zimmet PZ. Definition, diagnosis and classification of diabetes mellitus and its complications. Part 1: diagnosis and classification of diabetes mellitus provisional report of a WHO consultation. Diabet Med. 1998;15(7):539-53. Epub 1998/08/01. doi: 10.1002/(sici)1096-9136(199807)15:7<539::aid-dia668>3.0.co;2-s. PubMed PMID: 9686693.

2.	Corman VM, Eckerle I, Bleicker T, Zaki A, Landt O, Eschbach-Bludau M, et al. Detection of a novel human coronavirus by real-time reverse-transcription polymerase chain reaction. Euro Surveill. 2012;17(39). Epub 2012/10/09. doi: 10.2807/ese.17.39.20285-en. PubMed PMID: 23041020.

3.	Huang P, Wang H, Cao Z, Jin H, Chi H, Zhao J, et al. A Rapid and Specific Assay for the Detection of MERS-CoV. Front Microbiol. 2018;9:1101. Epub 2018/06/14. doi: 10.3389/fmicb.2018.01101. PubMed PMID: 29896174; PubMed Central PMCID: PMC5987675.

4.	Association AD. Standards of Medical Care in Diabetes—2020 Abridged for Primary Care Providers. Clinical Diabetes. 2020;38(1):10-38. doi: 10.2337/cd20-as01.

**CERTIFICATE OF ENGLISH EDITING**
