# Supplementary material for: Telemedicine management of type 2 diabetes mellitus in obese and overweight young and middle-aged patients during COVID-19 outbreak: A single-center, prospective, randomized control study
Source: PLoS One. 2022 Sep 29;17(9):e0275251. doi: 10.1371/journal.pone.0275251 (PMC9522303; doi:10.1371/journal.pone.0275251)
Supplement: Attachment — Submitted filename: Response to Reviewers.docx [file pone.0275251.s004.docx]

**Responds to the reviewer’s comments:**

- **Reviewer #1:** The author managed to answer to all the issue I raised. The paper has much improved and can now be further processed for publication

**Response:** It is a great honor to receive your help, and please accept my best thanks.

- **Reviewer #3:** The authors responded to reviewers comments. Finally I ask to improve the english form of the text. Please submit the revised form for a possible publication in the journal .

**Response:** Thank you for helping us a lot, your suggestion is of great help to us. We again invited a native English speaker to revise the language of the article. We put the new “**CERTIFICATE OF ENGLISH EDITING**” at the end of this article.
