# Supplementary material for: Telemedicine management of type 2 diabetes mellitus in obese and overweight young and middle-aged patients during COVID-19 outbreak: A single-center, prospective, randomized control study
Source: PLoS One. 2022 Sep 29;17(9):e0275251. doi: 10.1371/journal.pone.0275251 (PMC9522303; doi:10.1371/journal.pone.0275251)
Supplement: Attachment — Submitted filename: Response to Reviewers.docx [file pone.0275251.s005.docx]

**Responds to the reviewer’s comments:**

1. **Add p values to table 1.**

**Response:** We have added p values to Table 1.

2. **For all tables, add space between mean /median and the open parentheses. Add space after comma.**

**Response:** Thank you very much for the reminder! In Tabel 1 and Table 2, we have added space between mean /median and the open parentheses, and we have changed the expression of the median (IQR) to be consistent with that in "Result".
